# Cap‐to‐bell stage molar tooth morphogenesis occurs through proliferation‐independent sulcus sharpening and condensation‐associated tension in the dental papilla

**DOI:** 10.1111/joa.14187

**Published:** 2024-12-20

**Authors:** Claire Piper, Jeremy B. A. Green

**Affiliations:** ^1^ Centre for Craniofacial Regeneration and Biology King's College London, Guy's Hospital London UK

**Keywords:** cusp, enamel knot, molar, morphogenesis, sulcus, tooth

## Abstract

The anatomy of molar teeth is important both functionally for chewing food and in evolutionary studies as a well‐preserved species marker in the fossil record. Molar teeth begin to develop their characteristic biting‐surface shape of cusps (peaks) and sulci (valleys) at the bell stage, when corresponding folds in the dental epithelium become apparent. Theories about the developmental mechanisms of cusp and sulcus morphogenesis have hitherto largely focused on the non‐proliferating nature of the secondary enamel knots (EKs) at the cusp tips. EKs have been thought to direct cusp/sulcus formation by stimulating proliferative growth of the surrounding epithelium which, being confined within a capsule of condensed mesenchyme, bends by mechanical buckling. Here we show, using explant inhibition and cut‐and‐recoil experiments, that cap‐to‐bell morphogenesis is largely proliferation‐independent (sulcus sharpening entirely so) and that tension in the mesenchyme of the dental papilla, immediately sub‐adjacent to the cusps, rather than compression by the mesenchyme surrounding the whole structure, is what holds the structure in shape. Fine mapping of the degree of condensation shows that it is highest in the mesenchyme of the dental papilla and becomes progressively more focused to the cusp regions, consistent with a key role in cusp shaping. Together these findings overturn the prevailing models of molar morphogenesis, including both cusp and sulcus formation.

## Graphical abstract

Experiments in this article show that the development of the mouse molar tooth from cap‐stage to bell‐stage is not proliferation‐dependent, nor does it rely on confinement by condensing mesenchyme. Instead, morphogenesis is associated with condensation and tension in the mesenchyme of the dental papilla subadjacent to the forming cusps and sulcus.

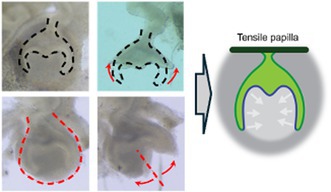

## 1 INTRODUCTION

Teeth are an outstanding example of ‘form follows function’ in biology, and they provide a link between diet and evolution, with the highly specific forms of the biting surface being a key feature. The development of the tooth is a long‐standing model for investigating morphogenesis. It has been a paradigm for the study of reciprocal epithelial–mesenchymal signalling in organogenesis (Yu & Klein, 2020). However, the mechanocellular mechanisms of tooth morphogenesis have received less attention.

Recent work has begun to shed light on the range of morphogenetic mechanisms that are involved in the early developmental stages of the tooth germ. Initial progression from the flat dental epithelium through to placode and bud stage involves cell‐on‐cell migratory behaviours termed vertical telescoping (Li et al., 2020) and canopy contraction (Panousopoulou & Green, 2016). Progression from bud to cap stage requires basal constriction in a population of epithelial cells on either side of the primary enamel knot (Yamada et al., 2019), the non‐proliferating epithelial signalling centre in the middle of the molar. Following cap stage, the molar tooth progresses to the bell stage in which the shape of the tooth, including the evaginated cusps (peaks) flanking a central sulcus (valley), become apparent in the shaping of the dental epithelium. Positioning of the cusps corresponds to the placement and size of secondary enamel knots (SEKs) (Thesleff et al., 2001), even when these are perturbed experimentally (e.g. by mechanical constraint (Renvoise et al., 2017) or heterotypic re‐patterning (Cai et al., 2007)). Here we investigate cap‐to‐bell physical morphogenesis mechanisms.

The prevailing idea in the literature for the generation of tooth cusps and sulci at bell stage has long been (Butler, 1956) that they form as the result of differential rates of proliferation between the enamel knots and the surrounding tissue. The varying rates of proliferation in the developing tooth have long been known and used to support this hypothesis (Jernvall et al., 1994; Salazar‐Ciudad & Jernvall, 2010). The primary and secondary enamel knots can be distinguished from surrounding epithelium by their lack of proliferation. Meanwhile, they produce signals that stimulate cell division of the surrounding tissue. Thus, both the non‐enamel knot epithelium and the underlying mesenchyme are highly proliferative during cap‐to‐bell stage morphogenesis (Ishida et al., 2011; Matalova et al., 2010; Morita et al., 2016; Obara & Lesot, 2007). As well as undergoing extensive proliferation, the developing tooth germ epithelium is constrained within a mesenchymal dental capsule consisting of the outer dental epithelium and surrounding mesenchyme. Morphogenesis of the tooth has therefore been theorised as being the consequence of what might be termed ‘buckling by proliferation under confinement’ (BPUC): proliferating epithelium within the confines of a dental capsule is forced to fold to relieve compressive stress (Marin‐Riera et al., 2018; Takigawa‐Imamura et al., 2015). Computational models generate the observed cusp/sulcus shapes by first establishing or assuming the SEK pattern (e.g. Salazar‐Ciudad & Jernvall, 2010) and then letting the differential proliferation drive BPUC (Marin‐Riera et al., 2018, Takigawa‐Imamura et al., 2015). As well as differential proliferation, the buckling‐by‐confined‐growth model has been supported by the finding that removal of the mesenchyme at E14.5 causes lateral splaying of the cervical loops, demonstrating that the width of the structure relies on the presence of the mesenchyme (Marin‐Riera et al., 2018; Morita et al., 2016).

However, it has recently been shown that the bud‐to‐cap stage folding of the epithelium occurs in the absence of proliferation (Yamada et al., 2019), demonstrating that—at least for this stage—the BPUC mechanism cannot apply. Moreover, the bud‐to‐cap morphogenesis is arrested by an inhibitor of focal adhesion kinase (FAK). FAK is associated with basal constriction in the folding of the neural tube at the midbrain‐hindbrain boundary in zebrafish (Gutzman et al., 2018) suggesting that an epithelium‐autonomous basal constriction mechanism is responsible for epithelial bending at the bud‐to‐cap transition in the molar tooth.

Here we show that cap‐to‐bell morphogenesis is mostly, but not entirely, proliferation independent, suggesting that—as at the bud‐to‐cap stage—differential proliferation and BPUC play, at most, a minor role. We further show that cusp formation is locally autonomous and relies on tension in the underlying papilla mesenchyme rather than confinement by the capsular mesenchyme. We further show that condensation in the papilla is much greater than in the surrounding mesenchyme, consistent with a role of sub‐adjacent condensation in cusp formation.

## 2 METHODS

### 2.1 Animals

Animals were handled under UK Home Office licensing and King's College London Ethics Committee approval. Pregnant wild‐type Crl:CD1(ICR) Cd‐1 mice were euthanised by cervical dislocation and embryos staged by weight and morphology (Peterka et al., 2002).

### 2.2 Ex vivo explant culture, treatment and dissection

Tooth germ slices from E15.5 mandibular molars were obtained according to previously described protocols (Alfaqeeh & Tucker, 2013). Briefly, mandibles were isolated from the embryo and sliced into 250 μm frontal sections using a McIlwain Tissue Chopper. Slices were then transferred to polyethylene terephthalate (PET) membranes (taken from BD Falcon™ cell culture inserts, pore size 0.4 μm, Cat. No 353095) and cultured on a steel mesh at an air/media interface. Media consisted of Dulbecco Modified Eagle Medium (DMEM) F12 (GIBCO, Invitrogen, Cat. No 11320033) supplemented with 1% penicillin/Streptomycin and 1% GlutaMAX™ (Gibco, Cat. No 10565018). Culture dishes were incubated at 37°C in 5% CO_2_. The medium was changed at 24‐h intervals.

For proliferation inhibition, explants were treated with Aphidicolin (2.0 μg/mL in dimethyl sulfoxide (DMSO); Santa Cruz Biotechnology) at E15.5 or E15.5+ 24 h time points and incubated for the following 24 h. Explants were pulsed with 10 μM 5‐ethynyl‐2′‐deoxyuridine (EdU) for 2 h after the 24 h inhibition. The slices were then fixed in 4% paraformaldehyde (PFA) for 3 h at room temperature (RT).

A fresh working solution of Gibco™ Dispase II (Cat. No [11510536]) was made on the day of the experiments at 50 U/ML. A dilution of 1.5 U/mL was used in culture media. Slices were cultured for 30–40 mins at 37°C in Dispase containing media to allow mesenchymal separation. Mesenchyme was then manually encouraged to separate from the epithelium using forceps and removed from the dish. Dispase containing media was quenched with fresh media, allowing further culture of the epithelium for up to 3 h before the shape was completely lost. To manually remove mesenchyme for cut and recoil experiments, 30G needles were used to cut tissue at various locations.

### 2.3 Immunofluorescence

For explant staining, antigen retrieval (DNA denaturation) was performed with 10 mM sodium citrate (pH 6) at 95°C for 30 min. The Click‐iT™ protocol (Click‐iT™ EdU kit (Invitrogen, Cat. No C10340)) was then applied before progressing to other antibody steps. Explants were washed x3 with Saponin‐based Permeabilisation and Wash Reagent and the permeabilised in 0.5% Triton X‐100 (T8787; Sigma) in PBS (PBST) for 30 mins. The explants were then incubated in the Click‐iT cocktail for 3 h at RT, then washed ×3 with wash buffer. To proceed with staining, explants were then blocked with 20% donkey serum (D9663; Sigma) in PBST for 60 min at RT. Explants were incubated with goat anti‐P‐cadherin (1:200, AF761; R&D Systems) at 4°C overnight. After six 1 h PBST washes, specimens were incubated at 4°C overnight with Alexa Fluor–conjugated secondaries (Life Technology). Nuclei were counterstained with DAPI (4′,6‐diamidino‐2‐phenylindole; Thermo Fisher Scientific, Cat. No. 62247) at 1:5000 dilution. Specimens were washed 6 times with PBST (1 h per wash) and then mounted on glass slides with 50% glycerol (Calbiochem, Cat. No. 356352) in PBS.

### 2.4 Nuclear staining for compaction

For nuclear spacing assays, thick sections were required to avoid grazing sections of cells. Embryos were collected at E15.5 and E16.5 and stage confirmed by weight. Mandibular explants were dissected and incubated at 55°C in 20% Type B Gelatin from Bovine skin (Sigma‐Aldrich, [Cat. No G9391]) in phosphate‐buffered saline (PBS), until samples were equilibrated. Mandibles were orientated, nose upwards, in moulds and solidified at 4°C. Blocks were trimmed and then re‐fixed in 4% PFA at 4°C with agitation for 3 days before washing to PBS. Blocks were cut using a Leica vibratome at 60 μm and slices containing tooth germs were collected in PBS in 24‐well plates for subsequent staining. Nuclear staining was performed by incubating with DAPI in PSB for 2 h at RT.

### 2.5 Imaging

Fluorescent images were taken on a Zeiss LSM980 confocal microscope. Images were taken using 20× (dry) and 40× (water immersion) objectives for the relevant experiments. For nuclear spacing analysis, images of slices were taken at 40× in Z‐stacks of 0.5 μm and started below the surface of the tissue to avoid surface artefacts.

### 2.6 Morphometrics and shape measurements

For analysis of explant shape, measurements of key axes of growth were taken before and after treatment. The width was measured across the tops of the cusps, and the sulcus depths were measured perpendicular to this line. The angle of the sulcus was calculated by drawing a circle whose diameter was half the length of the sulcus line and drawing an angle between the points where the circle met the epithelium and the tip of the sulcus.

For morphometric analysis, landmarks were placed in 11 reproducible positions on explants using tpsDig2w64 software (SB Morphometrics, freeware downloaded from www.sbmorphometrics.org) before being imported into MorphoJ software to allow comparisons between groups (Klingenberg, 2011). Procrustes fit was applied to remove scale as a variable and to generate the mean landmark positions for each group and statistically tested using the multiple permutation test (1000 permutations). Representative outlines were manually added to mean positions for visualisation.

### 2.7 Compaction maps

To atomically segment the nuclei for spacing analysis, StarDist, a deep‐learning tool (Schmidt et al., 2018) was used and trained on representative images from E15.5 and E16.5 sections. The nuclei were manually outlined to generate ‘ground truth’ for training. The training model was optimised for best performance as described by the developers. After segmenting the nuclei using Stardist, nuclear spacing maps were generated using previously developed code (Brock et al., 2016). In brief, centroids of each segmented nucleus were identified using Fiji (Schindelin et al., 2012), the nearest neighbours of each centroid were identified using the R ‘Deldir’ (Delaunay triangulation) package (Turner, 2023) distances between nearest neighbours and average distances to each cell's neighbours and its neighbours' neighbours were calculated. The average distance between each centroid and its neighbours was colour‐coded. Voronoi tessellation was used to tile the image into polygons (one per nucleus) and average neighbourhood distances were displayed using the assigned colours. To show mesenchymal compaction in the tooth papilla, epithelial regions were masked manually for clarity using Adobe Photoshop strictly according to their outlines in the original micrographs.

## 3 RESULTS

### 3.1 Molar morphogenesis at E15.5 is largely (but not completely) independent of proliferation

**FIGURE 1:**
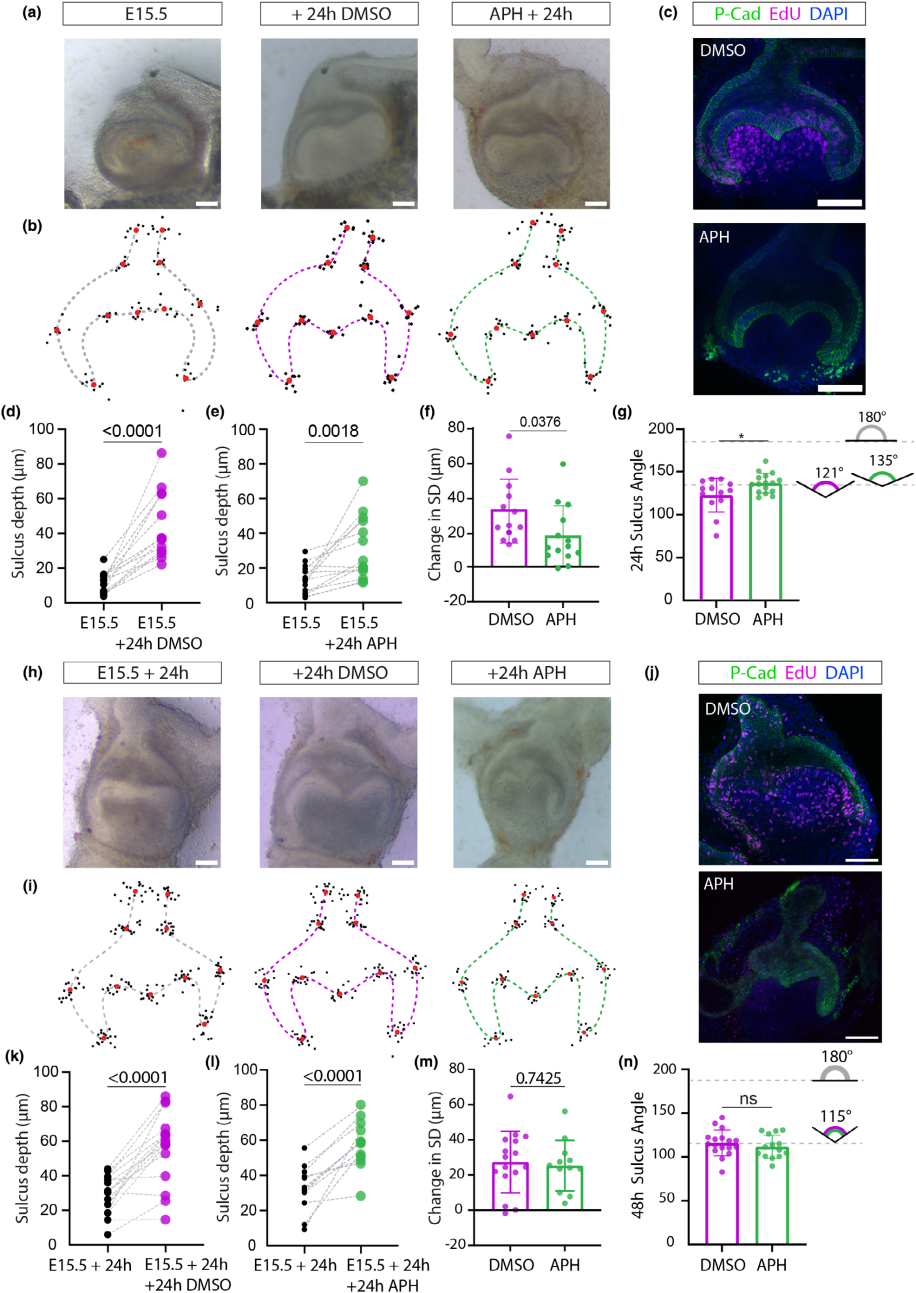
Proliferation inhibition by aphidicolin has small effects on morphogenesis at E15.5 + 24 h and none at E15.5 + 48 h. (a) Brightfield images of molar slices collected at E15.5 cultured with aphidicolin (APH) or DMSO (vehicle) for 24 h. (b, i) 11 landmarks placed along epithelial contour. Black dots represent individual sample landmark locations, red dots represent average. Dashed lines drawn manually for E15.5 (grey) DMSO (magenta) and APH‐treated (green) epithelium shapes (c, j) EdU incorporation marks cell divisions. Cultures were pulsed with EdU for 2 h before fixing and staining. EdU stain (magenta) is absent from APH cultures. P‐cadherin (green) marks tooth epithelium to visualise shape. DAPI nuclear counterstain (Blue). (d, e) Sulcus depth measured from the highest point of the cusps increases after 24 h in both controls (d) and with APH (e) but there is a significant reduction in the APH treated sulcus depth (f). (g) Sulcus angle is increased in APH treated explants at 24h (*p = 0.016, unpaired t‐test); DMSO (purple) APH (Green). (h) Brightfield images of molar slices collected at E15.5, cultured for 24 h before addition of Aphidicolin (APH) or DMSO (vehicle) and culture for a further 24 h. (k, l) Sulcus depth measured from the highest point of the cusps increases after 24 h in both controls (k) and with APH (l). (m) No significant difference between depth of sulcus in APH and controls (n) Sulcus angle is increased in APH treated explants at 24 h DMSO (purple) and APH (Green).

To test the hypothesis that cap‐to‐bell tooth morphogenesis is solely attributable to differential proliferation and consequent buckling by confinement, we inhibited proliferation using aphidicolin (APH) on embryonic day (E) 15.5 (early bell stage) molar explants. Given previous work had shown that aphidicolin cannot be applied to explants for more than 24 h (when the tissue integrity starts to break down (Yamada et al., 2019)) explants were imaged at 24 h and compared to controls (Figure 1). The cultures were pulsed with EdU during culture to confirm inhibition of proliferation (Figure 1c).

No proliferation occurred in the treated explants, and they were smaller than controls but, contrary to the buckling‐by‐confined‐growth model, their epithelial morphogenesis appeared largely normal (Figure 1a). To quantify overall shape differences between the proliferation‐inhibited and control explants we used geometric morphometrics (MorphoJ) (Figure 1b). We found no statistically significant difference between the shapes determined by defined landmarks (*p* = 0.93, multiple permutation *T* ^2^ test).

However, we were concerned that high shape variability in some parts of the explants might be masking consistent differences in other parts. Therefore, to further analyse the shape changes, we quantified changes in the depth of the sulcus (measured relative to the highest point of the cusps). Sulcus depth was zero at E15.5 explantation and increased after 24 h culture in both APH‐treated and control explants (paired *t*‐tests, *p* < 0.0001, *p* = 0.0018) but significantly less after 24 h culture in the APH‐treated cultures compared to controls, on average 32 μm in controls and 16 μm in APH‐treated explants (unpaired *t*‐test, *p* = 0.038) (Figure 1d,e). We also analysed the sharpness (subtended angle) of the sulcus and found that the APH‐treated explants showed a slightly, but statistically significantly, widened angle at 24‐h compared to the more acute angle in the control explants (Figure 1f). Together, these results indicated that proliferation is probably an important but not sole factor in generating the sharpness and depth, relative to the cusps, of the central molar sulcus at E15.5.

### 3.2 Sulcus sharpening progresses in the absence of proliferation

From E16.5 to E17.5, the molar sulcus sharpens, and the cusps rise relative to it. Due to the increased mineralisation at E16.5, slice cultures are somewhat more difficult to isolate at this stage, but since germs cultured from E15.5 for 48 h reliably recapitulate developmental morphogenesis to E17.5 (data not shown and (Alfaqeeh & Tucker, 2013)), we collected slices at E15.5 and cultured them without treatment for 24‐h, then applied APH for a further 24 h to inhibit proliferation to the later developmental stage. Comparably to the earlier stage, the growth in size was inhibited (consistent with inhibition of cell proliferation) but the gross shape of the explants after 24 h in culture appeared largely unaffected by APH treatment: both treated and untreated explants developed a sharp, V‐shaped, central sulcus and rounded cusps with no statistically significant difference in shape (multiple permutation *T* ^2^ test, *p* = 0.23) (Figure 1h,j). Sulcus depth again significantly increased with or without APH (paired *t*‐tests, *p* < 0.0001 for both) but, unlike the earlier stage, there was no difference in this depth increase between the treated and control explants (unpaired *t*‐test, *p* = 0.74) (Figure 1k–m). Thus, sulcus deepening can proceed fully at E16.5 in the absence of proliferation. Consistent with this, we found no difference between the control and treated explants in sulcus angle (unpaired *t*‐test, *p* = 0.41) (Figure 1n). Thus, although the initial generation of the sulcus shape (E15.5 + 24 h) is partially dependent on proliferation, molar morphogenesis at late bell stage (E16.5 + 24 h) appears to be proliferation independent.

### 3.3 Cervical loop orientation depends on tension in the papilla more than mesenchyme enclosure

**FIGURE 2:**
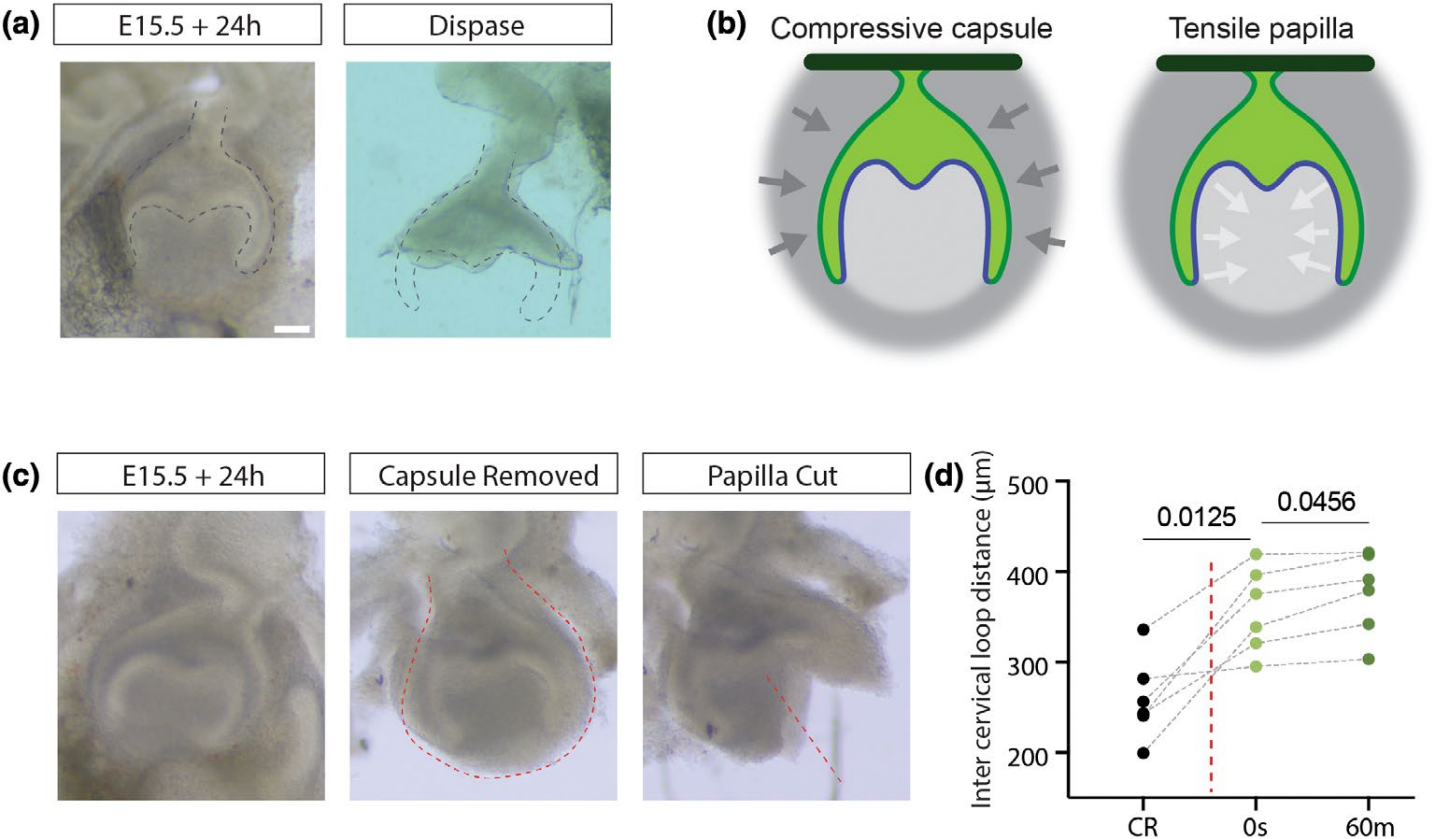
Cervical loops and sides of the cusps are held in place by papilla tension not capsule compression. (a) Brightfield images of an E15.5 molar tooth explant cultured for 24 h before and after mesenchyme removal by dispase treatment as in (Marin‐Riera et al., 2018; Morita et al., 2016) showing ‘splaying’ of released cervical loops. (b) Schematic illustrating alternative interpretations of the results shown in (a). (c) Brightfield images of a molar tooth slice intact, with the outer capsule manually removed showing unchanged shape, and with the mesenchyme of the dental papilla cut vertically showing recoil from the line of the cut. (d) Quantification of multiple experiments of the kind illustrated in (c) showing an initial fast recoil upon cutting followed by a slower recoil after 60 min incubation.

As well as proliferation, models of BPUS require constriction from the dental capsule, a putatively confining zone of condensed mesenchyme that would hold the cervical loops into place by compression and would force buckling of the enclosed epithelium as proliferation increases its planar area. If mesenchymal constraint is providing force to push the epithelium into its curved contour, it would follow that removal of the constraining tissue could immediately release this elastic tension and flatten the epithelium, and it has indeed been previously shown that at E14.5 the cervical loops immediately spring outwards upon complete removal of mesenchyme (Marin‐Riera et al., 2018; Morita et al., 2016). To investigate this idea at later bell stages, we separated the epithelium from the mesenchyme using dispase at E15.5 + 24 h culture (approximately equivalent to E16.5). We confirmed that, as at the earlier stage, mesenchyme is required to maintain the cervical loops in position but noticed that the sulcus shape of the inner dental epithelium was maintained upon mesenchymal release (Figure 2a).

As dispase removes the entire mesenchyme, we could not distinguish force on the cervical loops being due to an external compressive force from the capsular mesenchyme (i.e. the condensed mesenchyme outside the cervical loops) or internal tensile one within the dental papilla (Figure 2b). To distinguish these two, we manually removed the capsular mesenchyme and found that the tooth epithelium, including the cervical loops, held its shape and continued to develop normally. This suggested that the cervical loops are held in place by tension in the papilla mesenchyme. To test this, we made cuts through the middle of the papilla after the external capsule had been removed. This caused an immediate springing outwards of the cervical loops (Figure 2c,d), significantly increasing distance between them (*n* = 6, Tukey test following one‐way ANOVA, *p* = 0.013). This distance further increased over the following hour after cutting, indicating a viscoelastic relaxation (Figure 2d). Both the degree of further relaxation and the final position of the cervical loops represented significant changes from the uncut shape (*p* = 0.046 and *p* = 0.012 respectively).

These experiments together tend to disprove models of tooth germ folding that require an external compressive structure (Takigawa‐Imamura et al., 2015) and show that the force required for correct orientation of the cervical loops comes from tension within the dental papilla.

### 3.4 Cusps can elevate in the absence of an intact capsule

**FIGURE 3:**
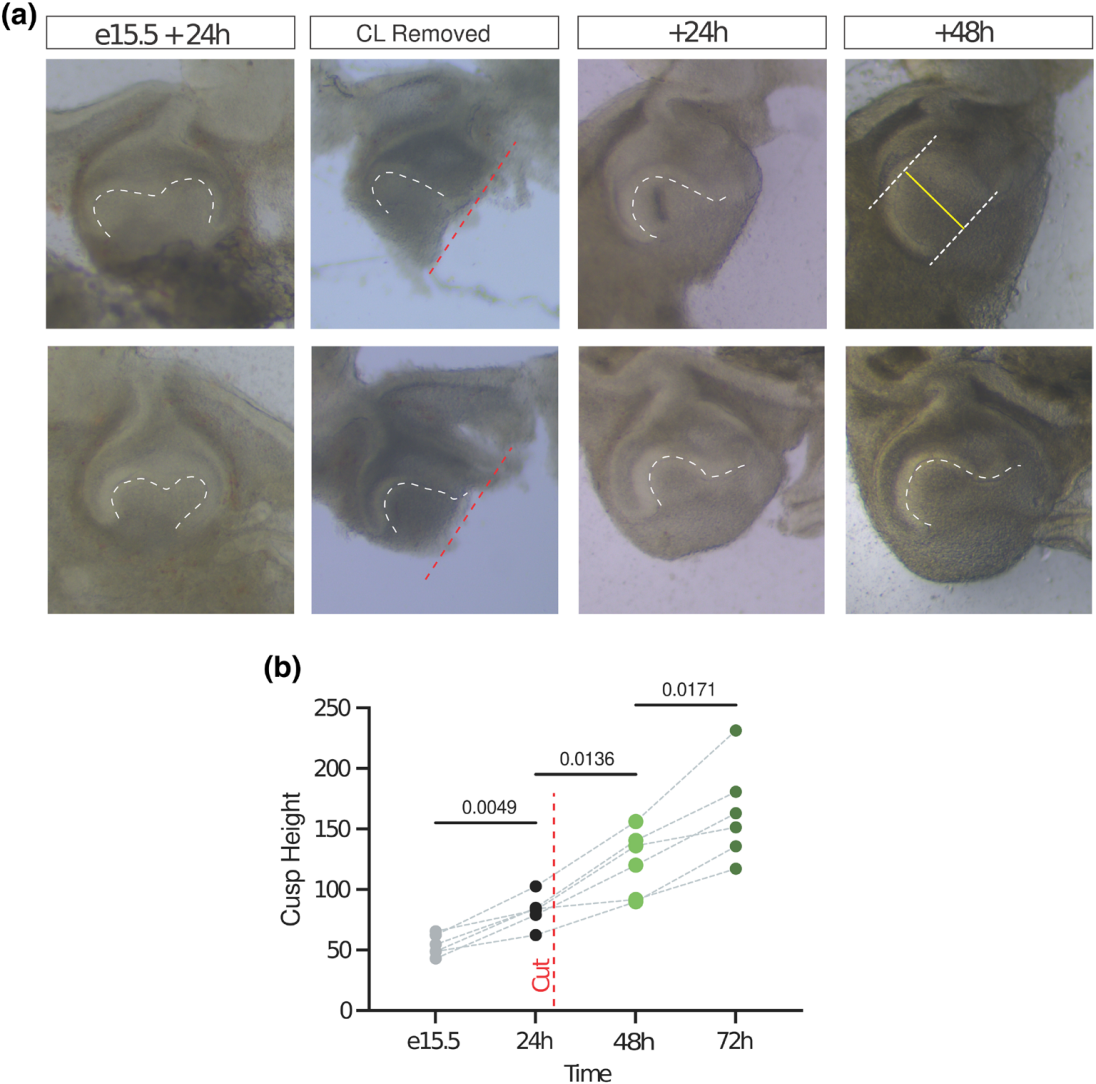
Elevation of the cusps is autonomous to each cusp and must rely on epithelium or sub‐adjacent mesenchyme. (a) Brightfield images of two example E15.5 molar slice explants cultured for 24 h intact and then trimmed to leave a single cusp region, then after 24‐ and 48‐h further culture showing continued cusp heightening. (b) Quantification of multiple experiments of the kind illustrated in (a) showing ongoing heightening of isolated single cusps.

As a further test of the hypothesis that cusp morphogenesis depends on mechanical confinement of the dental epithelium by surrounding mesenchyme, we cultured slice explants from which one entire cervical loop and surrounding mesoderm on one side had been removed (Figure 3a). After 24 and 48 h, we measured the height of the cusp region relative to a line drawn between the remaining cervical loop tip and the sulcus. We found that despite the loss of integrity of the epithelial structure and mesenchymal capsule, the cusp height continued to increase significantly (Figure 3a,b). Although some mesenchyme remained or regrew below the cusp, which may have contributed to morphogenesis, these data suggested that a global constraint is not required to generate force for increasing curvature, implying that morphogenetic forces are localised to at most a half‐papilla scale, but potentially to much smaller scales such as the basal ends of individual epithelial cells or tensile mesenchymal cells immediately underlying them.

### 3.5 Mesenchyme undergoes compaction below the forming cusps

**FIGURE 4:**
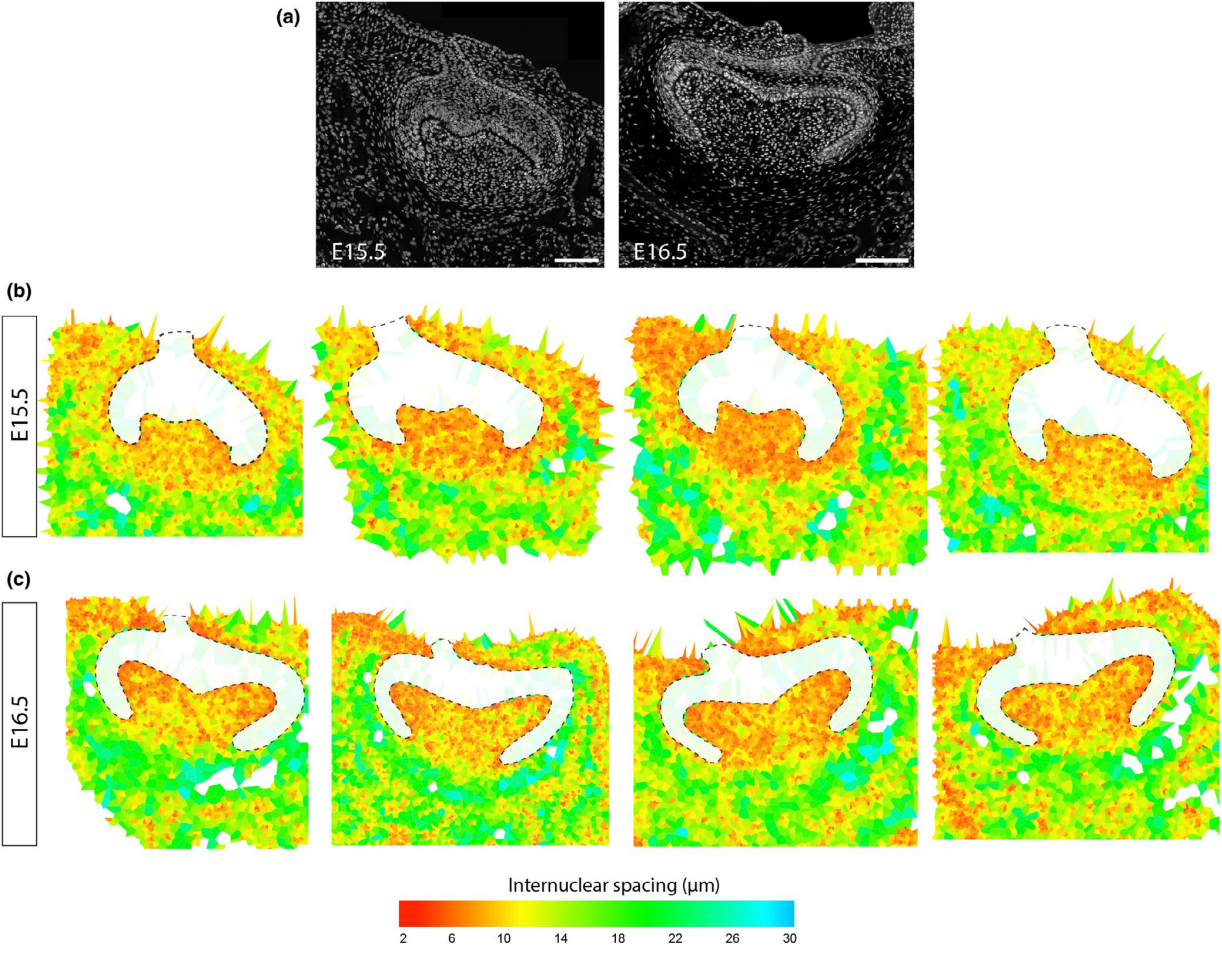
Internuclear distance mapping reveals dental mesenchyme condensation is primarily in the papilla and increasingly focused to sub‐cuspal regions. (a) Confocal images of DAPI‐stained molar tooth sections at the stages indicated of the kind used for internuclear distance mapping. (b) Examples of four different molar tooth maps at each of E15.5 and E16.5 showing the internuclear spacing averaged locally for each nucleus, its neighbours and neighbours' neighbours. For clarity, epithelial regions (white) are not shown but outlined by the dashed lines.

One mechanism by which the papilla could exert tension to both direct the cervical loops and bend the cusp epithelium is condensation. To capture condensation, we used a cell‐to‐cell distance averaging method (previously developed to map packing density in palate epithelium (Economou et al., 2013)). Mapping cell spacing in this way showed that at E15.5 the mesenchyme surrounding the tooth is rather loosely packed while the dental papilla mesenchyme is relatively condensed (Figure 4). Some E15.5 specimens showed slightly less condensation, suggesting that this stage might be when condensation is in progress. The region of condensation extended below the ‘neck’ of the papilla (literally the ‘cervix,’ that is, below an imaginary line between the tips of the cervical loops) at this stage (E15.5, Figure 4b). Given cuts to the internal papilla led to the spring up of the cervical loops, it is likely that this compaction leads to increased tension throughout this region.

At E16.5, the curvature of the cusps was more apparent. The dental papilla remained more compact than the surrounding mesenchyme, but compaction was restricted to the region between the cervical loops. However, the compaction was less uniform in the dental papilla than at E15.5, both locally more non‐uniform but also consistently (albeit subtly) higher immediately under the elevating cusps. These findings are consistent with both a general role for condensation in the papilla directing the cervical loops and a later specific role in driving or enhancing the formation of the molar cusps.

## 4 DISCUSSION

In this study, we have demonstrated the continued progression of epithelial bending to generate the bell‐shaped tooth in slice explants in the absence of cell proliferation. The proliferation arrest was complete in that no further incorporation of EdU was detected, although since aphidicolin arrests only at S‐phase, cells past that phase would have continued to grow and undergo mitosis. This means that some tissue growth will have occurred in these experiments. Thus, these experiments do not rule out a role for differential growth entirely, but rather show that most of the development in each of the 24‐h periods tested is proliferation‐independent.

We confirmed others' previous findings that mesenchyme shapes the sides of the developing molar, the outer dental epithelium and the sides of the inner dental epithelium that together form the cervical loops (future tooth roots). However, our experiments shift the emphasis from compression from the capsule outside to tension from the papilla inside as the essential mechanical influence. Where does the notion of capsular compression come from? It is true that the histological appearance of the capsule is of mesenchymal condensation and, within the condensation, of circumferential elongation of cell nuclei as though stretched around the tooth structure. Moreover, there is good evidence that, at least at slightly later stages, external mechanical constraint limits overall tooth growth (Alfaqeeh et al., 2013) and can modify the pattern of cusps (Renvoise et al., 2017). However, our results suggest that the latter may be due to changes in signalling (changing the boundary conditions of a reaction–diffusion patterning field) rather than direct mechanical bending of the epithelium. We propose that condensation and mechanical forces in the capsule are quantitatively less than those in the papilla and not so mechanically critical in the formation of cusp shape as papilla mesenchyme.

The idea that mesenchymal condensation can drive evagination of immediately overlying epithelium is not new. Premature activation of Wnt signalling in the molar tooth causes both condensation and evagination (Kim et al., 2021) while initiation of villus formation in the gut is associated with condensations of a population of underlying mesenchymal cells (Huycke et al., 2024). Thus, the mechanistic question is whether the condensation is truly the driver in the tooth and, if so, how does it work? Alternatively, is the mesenchymal condensation merely correlated with a basal narrowing/constriction process in the epithelium to drive cusp formation? It is also hard to explain sulcus sharpening as a consequence of a mesenchymal condensation mechanism. Sulcus formation has in general received scant attention in the literature, and its development, function and evolution deserve further investigation.

Together our findings overturn the prevailing understanding of tooth cusp morphogenesis by differential proliferation and replace it with a model in which active local contractility in papillar mesenchyme and in the epithelium itself generate tooth cusps and sulci.

## AUTHOR CONTRIBUTIONS

**Claire Piper:** concept/design, acquisition of data, data analysis/interpretation, drafting of the manuscript, critical revision of the manuscript. **Jeremy B. A. Green:** concept/design, data analysis/interpretation, drafting of the manuscript, critical revision of the manuscript.

## Data Availability

The data supporting this article are openly available from the King's College London research data repository, KORDS, at https://doi.org/10.18742/27733866.
